# Loss of intra-islet heparan sulfate is a highly sensitive marker of type 1 diabetes progression in humans

**DOI:** 10.1371/journal.pone.0191360

**Published:** 2018-02-07

**Authors:** Charmaine J. Simeonovic, Sarah K. Popp, Lora M. Starrs, Debra J. Brown, Andrew F. Ziolkowski, Barbara Ludwig, Stefan R. Bornstein, J. Dennis Wilson, Alberto Pugliese, Thomas W. H. Kay, Helen E. Thomas, Thomas Loudovaris, Fui Jiun Choong, Craig Freeman, Christopher R. Parish

**Affiliations:** 1 Department of Immunology and Infectious Disease, The John Curtin School of Medical Research, The Australian National University, Canberra, Australian Capital Territory, Australia; 2 Department of Internal Medicine III, Carl Gustav Carus Medical School, Technical University of Dresden, Dresden, Germany; 3 Department of Endocrinology, The Canberra Hospital, Woden, Australian Capital Territory, Australia; 4 Diabetes Research Institute, Departments of Medicine, Microbiology and Immunology, University of Miami Miller School of Medicine, Miami, Florida, United States of America; 5 St Vincent’s Institute of Medical Research, Fitzroy, Melbourne, Victoria, Australia; 6 Department of Cancer Biology and Therapeutics, The John Curtin School of Medical Research, The Australian National University, Canberra, Australian Capital Territory, Australia; Children's Hospital Boston, UNITED STATES

## Abstract

Type 1 diabetes (T1D) is an autoimmune disease in which insulin-producing beta cells in pancreatic islets are progressively destroyed. Clinical trials of immunotherapies in recently diagnosed T1D patients have only transiently and partially impacted the disease course, suggesting that other approaches are required. Our previous studies have demonstrated that heparan sulfate (HS), a glycosaminoglycan conventionally expressed in extracellular matrix, is present at high levels inside normal mouse beta cells. Intracellular HS was shown to be critical for beta cell survival and protection from oxidative damage. T1D development in Non-Obese Diabetic (NOD) mice correlated with loss of islet HS and was prevented by inhibiting HS degradation by the endoglycosidase, heparanase. In this study we investigated the distribution of HS and heparan sulfate proteoglycan (HSPG) core proteins in normal human islets, a role for HS in human beta cell viability and the clinical relevance of intra-islet HS and HSPG levels, compared to insulin, in human T1D. In normal human islets, HS (identified by 10E4 mAb) co-localized with insulin but not glucagon and correlated with the HSPG core proteins for collagen type XVIII (Col18) and syndecan-1 (Sdc1). Insulin-positive islets of T1D pancreases showed significant loss of HS, Col18 and Sdc1 and heparanase was strongly expressed by islet-infiltrating leukocytes. Human beta cells cultured with HS mimetics showed significantly improved survival and protection against hydrogen peroxide-induced death, suggesting that loss of HS could contribute to beta cell death in T1D. We conclude that HS depletion in beta cells, possibly due to heparanase produced by insulitis leukocytes, may function as an important mechanism in the pathogenesis of human T1D. Our findings raise the possibility that intervention therapy with dual activity HS replacers/heparanase inhibitors could help to protect the residual beta cell mass in patients recently diagnosed with T1D.

## Introduction

Type 1 diabetes is an autoimmune disease which destroys the insulin-producing beta cells of pancreatic islets [1–3]. T lymphocytes have been detected in islet-associated inflammation (insulitis) strongly supporting a role for T cell-mediated autoimmune responses in the disease process [4–6]. However, recent clinical trials testing the blockade of T cell activation and function as well as cytokine-based strategies for immunomodulation in patients with new-onset type 1 diabetes have resulted in only limited therapeutic benefit, with a slower decline in insulin secretion and a modest impact on insulin requirement and disease progression [1, 7–10]. These outcomes have stimulated other avenues of research to better understand the pathogenesis of the disease and to develop more effective intervention strategies. Cadaver donor or archival human pancreas specimens [11, 12] and live donor pancreas and blood specimens [13–16] have been used to investigate islet-infiltrating leukocytes in insulitis lesions [4, 17, 18], the role of the extracellular matrix (ECM) in regulating the intra-islet entry of leukocytes into human islets [19, 20], the modulation of peripheral blood neutrophil levels [15, 16] and the contribution of enteroviral infection of beta cells as a potential trigger for leukocyte recruitment [21, 22]. In addition, it has become increasingly evident that the residual beta cell mass at diagnosis is much more significant than previously estimated [3, 23], highlighting the potential for therapeutic strategies to safeguard these viable beta cells and preserve their function [7, 9]. To this end, therapeutic interventions have largely focused on directly suppressing the autoimmune response in T1D and little attention has been devoted to better understanding the intrinsic requirements for beta cell survival. In this study we investigated a role for intracellular heparan sulfate (HS), a sulfated glycosaminoglycan, as a requirement for the survival of human beta cells and as a marker of beta cell damage in human T1D, identifying HS preservation as a possible novel therapeutic strategy for beta cell protection and preventing T1D progression.

HS is a linear polysaccharide composed of repeating dissacharides (consisting of glucosamine and uronic acid) and is covalently attached to core proteins, forming heparan sulfate proteoglycans (HSPGs). HSPGs are classified by their specific core protein and are conventionally localized in extracellular matrix (ECM; e.g., collagen type XVIII (Col18), agrin) and on the surface of cells (e.g., syndecans, glypicans). Their HS side chains act as adhesion molecules and as reservoirs for chemokines, cytokines and growth factors [24, 25]. Perlecan, a large HSPG, is found in basement membranes (BMs), including the peri-islet BM, and helps to prevent cell invasion [26]. We have previously identified the unusual localization of HS and the HSPGs Col18 and syndecan-1 (Sdc1), inside mouse beta cells [27, 28]. HS in beta cells has been shown to have diverse functions which are regulated largely by the HS sulfation pattern and related, in some instances, to specific HSPG core proteins. Of significance, highly sulfated HS was reported to be essential for the survival of primary beta cells and to provide protection from oxidative damage [27]. In support, desulfation of HS in the rat INS1 beta cell line increased the sensitivity of the beta cells to hydrogen peroxide-induced damage [29]. Furthermore, by depleting beta cell HS in mice, Takahashi et al demonstrated a critical role for HS in islet development and beta cell function [30]. Specifically, beta cell HS, 3-*O*-sulfation of HS and the HSPG syndecan-4 have been shown to play important roles in the secretion of insulin by mouse beta cells or beta cell lines [30–32]. However, little is known about HS and its roles in human beta cells.

HS is more widely recognized for its extracellular distribution in the ECM and basement membranes, regulating in particular, cell invasion, cell migration and inflammation [25, 33–35]. Heparanase, the only mammalian endoglycosidase, degrades HS and plays a critical role in permitting cells, including leukocytes, to traverse basement membranes and to migrate into underlying tissues [25]. Heparanase is produced by various leukocytes and has also been shown to be expressed by endothelial cells and/or susceptible tissues in acute and chronic inflammatory disease models, e.g., acute pancreatitis, ulcerative colitis, glomerulonephritis [36–38], as well as in diabetes-related complications e.g., nephropathy and retinopathy [39–41]. We have previously found that the unusually high levels of HS in islet beta cells and the localisation of the HSPG perlecan in the peri-islet basement membrane render islet beta cells particularly vulnerable to heparanase-mediated damage. During T1D development in non-obese diabetic (NOD) female mice, islet HS progressively declined and disruption of the peri-islet BM correlated with the expression of the HS-degrading enzyme heparanase by infiltrating leukocytes in the insulitis lesions [26, 27]. Significantly, treatment of NOD mice with the heparanase inhibitor/HS replacer PI-88, reduced the incidence of diabetes by ~50% and preserved intra-islet HS [27]. These observations provide strong support for heparanase-mediated depletion of islet HS as an important mechanism in the pathogenesis of T1D [42].

In this study we examined the clinical relevance of HS for human beta cell survival and as a target during T1D disease development in humans. Our findings confirm the presence of high levels of intra-islet HS and HSPG core proteins in normal human islets and support a unique role for HS in human beta cell survival; furthermore, we demonstrate that beta cell HS is lost before insulin in human T1D and is a sensitive marker of disease progression.

## Methods

### Human samples

Paraffin sections of formalin-fixed human pancreas specimens from non-diabetic organ donors (n = 8), T1D donors with insulin-positive (Ins+) islets (n = 8) and T1D donors with insulin-negative islets (n = 10) were obtained from the JDRF Network for Pancreas Organ Donors with Diabetes (nPOD) [11]. nPOD pancreases were procured with consent and under the University of Florida IRB review; all nPOD human pancreas samples were de-identified and the donor T1D/non-T1D status was accessed anonymously. T1D pancreases with insulin-containing islets (#6046, #6052, #6069, #6070, #6084, #6198, #6209, #6212) were obtained from 5–22.9 year-old donors at 0.25–8 years after diagnosis. The control donors (#6012, #6075, #6094, #6096, #6102, #6104, #6129, #6134) were 2.9–68 years of age. Isolated human islets were obtained from the Tom Mandel Islet Transplant Program at St Vincent’s Institute of Medical Research (SVI), Melbourne (Australia) and from the Integrated Islet Distribution Program (City of Hope National Medical Center, Duarte, CA; http://www.iidp.coh.org). Documented consent was provided for the use of human islets for research, the donors were de-identified and batches of isolated human islets together with analytical data were labeled with a SVI number or Lot number, respectively, to ensure the anonymity of the donor. These procedures were carried out with review and approval by appropriate regulatory authorities. Islets with 86.8±7.9% (n = 10) purity were used at 1–7 days post-isolation. The shipping time varied between ~7 hours (national) to ~35 hours (international). Formalin-fixed isolated human islets were also provided by the Department of Medicine, Technical University of Dresden (Dresden, Germany). Research using isolated human islets and pancreas specimens was approved by the Australian National University (ANU) Human Research Ethics Committee (protocols 2008/536 and 2014/689).

### Preparation and culture of isolated human islet cells

Isolated human islets were dispersed into single cells using Accutase (Milllipore, Temecula, CA), ~1500–2000 islet equivalents/ml (https://dx.doi.org/10.17504/protocols.io.kwwcxfe). 2.0–6.5 x 10^4^ islet cells were transferred to individual wells of a 96 well culture plate (CELLSTAR, Greiner Bio-one, Frickenhausen, Germany) for immediate staining for flow cytometry analysis or for culture in RPMI 1640 medium (Sigma-Aldrich, St Louis, MO) supplemented with 10% fetal calf serum (Sigma-Aldrich) and antibiotics (penicillin G (0.06 mg/ml; MP Biomedicals, Santa Ana, CA) /streptomycin (0.10 mg/ml; Sigma) /neomycin (0.10 mg/ml; Sigma)) [27] prior to staining.

### Culture of isolated human islet cells with HS mimetics

Isolated human islet cells were cultured in the presence (50 μg/ml) or absence of the HS mimetics heparin (a highly sulfated HS analogue from porcine intestinal mucosa; Celsus Laboratories, Cincinnati, OH) or FITC-heparin (see Supporting Information, S1 Appendix), BT548 (a glycol split low molecular weight heparin (LMWH; 3 kDa) lacking anti-coagulant activity [43, 44]) or PI-88 (phosphomannopentaose sulfate; Progen Pharmaceuticals Limited, Brisbane, Australia) [45] for 2 days in 5% CO_2_, 95% air at 37°C [27]. PI-88 is structurally distinct from BT548. BT548 is a LMWH derived from limited nitrous acid treated, glycol split (periodate-treated) heparin isolated from porcine intestinal mucosa and consists of a mixture of sulfated oligosaccharides containing uronic acid and glucosamine residues. In contrast, PI-88 is a mixture of highly sulfated monophosphorylated mannose oligosaccharides (predominantly phosphomannopentaose sulfate and phosphomannotetraose sulfate), derived from the extracellular phoshomannan of the yeast *Pichia holstii* [45]. In some studies islet cells were acutely treated with 30% H_2_O_2_ (Chem-Supply, Gillman, Australia) as a source of reactive oxygen species (ROS) for 5 min on day 0 or after culture for 2 days with/without HS mimetics.

### Flow cytometry

Beta cells were identified by staining with Newport Green (NG; 10 μmol/L; Invitrogen, Molecular Probes, Eugene, OR), a fluorescent probe that detects zinc in the insulin granules of beta cells [46]. Damaged and dying islet cells were assessed using 7-Aminoactinomycin (7AAD, 10 μg/ml; Life Technologies, Eugene, OR) or by Sytox green (31.25 nmol/L; Invitrogen, Molecular Probes) uptake (https://dx.doi.org/10.17504/protocols.io.kwwcxfe) [27]. For intracellular staining, isolated islet cells were fixed in 2% paraformaldehyde (Sigma-Aldrich) and permeabilized using 0.3% saponin (Sigma-Aldrich). The cells were stained with 10E4 mouse anti-human HS mAb (10E4, 1/50; Seikagaku, Tokyo, Japan or US Biological/Amsbio, Abingdon, UK), mouse anti-mouse Col18 mAb (1/50; Santa Cruz Biotechnol., Santa Cruz, USA) or the corresponding isotype control Ig (mouse IgM_κ_ or IgG_2bκ_; BD Biosciences, San Jose, CA) followed by goat anti-mouse Ig-R-phycoerythrin (1/100; Southern Biotech, Birmingham, AL)

(https://dx.doi.org/10.17504/protocols.io.kwzcxf6) [27]. The geometric mean fluorescence ratio (GMFR) was calculated by dividing the geometric mean fluorescence intensity (GMFI) of cells stained with primary mAb by the GMFI obtained with the relevant isotype control Ig [27]. Cells were analyzed using a BD LSRI flow cytometer and CellQuest™ Pro software (version 6.0; BD Biosciences).

### Histology and immunohistochemistry

For quantitative analyses of HS, HSPGs, insulin and glucagon localization in human islets, paraffin sections (4 μm thickness) of nPOD human pancreases and isolated human islets fixed in 10% neutral-buffered formalin were stained with hematoxylin and eosin (H&E) or by immunohistochemistry. Antigen retrieval for HS and Col18 was performed using 0.05% pronase (Calbiochem, Japan) [27, 28], whereas heat/citrate buffer (pH 6) was used for Sdc1 and heparanase [27, 28]. HS and HSPG core proteins were detected immunohistochemically using 10E4 anti-HS (1/5-1/10; https://dx.doi.org/10.17504/protocols.io.kvzcw76), anti-Col18 (1/100; https://dx.doi.org/10.17504/protocols.io.kvzcw76) and rat anti-mouse Sdc1 (CD138, 1/10; BD Biosciences) (https://dx.doi.org/10.17504/protocols.io.kv3cw8n) mAbs, with horseradish peroxidase-conjugated rabbit anti-mouse or anti-rat Ig (Dako, Carpinteria, USA). Heparanase was localized using the HP130 mouse anti-human heparanase mAb (1/5; Insight Biopharmaceuticals, Rehovot, Israel), biotinylated anti-mouse IgG (1/250) and avidin-biotin-complex (ABC reagent; PK-2200, Vector Laboratories, Burlingame, CA) (https://dx.doi.org/10.17504/protocols.io.kv4cw8w). Background staining was checked using the corresponding isotype control Ig and human pancreatic lymph node (PLN) was used as a positive control. Insulin and glucagon were detected using mouse anti-insulin (ascites; 1/250) or mouse anti-glucagon (ascites; 1/500) mAbs (Sigma-Aldrich) and biotinylated anti-mouse IgG/ABC reagent (https://dx.doi.org/10.17504/protocols.io.kv6cw9e). 3-amino-9-ethylcarbazole (AEC; Sigma-Aldrich) was used as the chromogen. Specimens were de-identified prior to morphometric analysis. Image J software with color deconvolution plugin was used for the quantitative analysis of the % of islet area stained [27, 28] in 7–10 islets/donor pancreas.

### Immunofluorescence microscopy

For colocalization studies, paraffin sections were treated with 0.05% pronase for antigen retrieval, blocked with 2% bovine serum albumin (BSA; Sigma)/phosphate buffered saline (PBS), incubated overnight (4° C) with 10E4 (anti-HS) mAb (1/10), washed and stained with AlexaFluor 488-goat anti-mouse IgM (Thermo Fisher, Rockford, IL, USA). The same sections were washed, incubated with rabbit anti-human glucagon IgG (Abcam, Cambridge, UK) or guinea-pig anti-insulin Ig (Dako, Santa Clara, CA, USA), washed and stained with Alexafluor 568-donkey anti-rabbit IgG or AlexaFluor 568-goat anti-guinea-pig IgG (Thermo Fisher) (https://dx.doi.org/10.17504/protocols.io.kvycw7w). The specificity of HS staining was checked on serial sections using IgM_κ_ isotype control (BD Biosciences), instead of 10E4 mAb, together with anti-glucagon or anti-insulin antibody. Nuclei were stained with DAPI (0.2 μg/ml; Sigma). Sections were imaged using an automated Axio Observer inverted fluorescence microscope (Zeiss; Göttingen, Germany). Merged images were prepared using ZEN (version 2.3) software (Zeiss).

### Statistical analyses

For comparisons between groups in immunohistochemical analyses, the 2-tailed, unpaired Student’s t test and Mann-Whitney test were used. One-way ANOVA with Bonferroni Multiple Comparisons test, non-parametric ANOVA (Kruskal-Wallis test) with Dunn’s Multiple Comparisons test, unpaired Student’s t-test or Mann-Whitney test were used to analyse flow cytometry data. P<0.05 was considered to be statistically significant.

## Results

### Distribution of intra-islet HS and HSPG core proteins in normal and diabetic human pancreas

**Fig 1:**
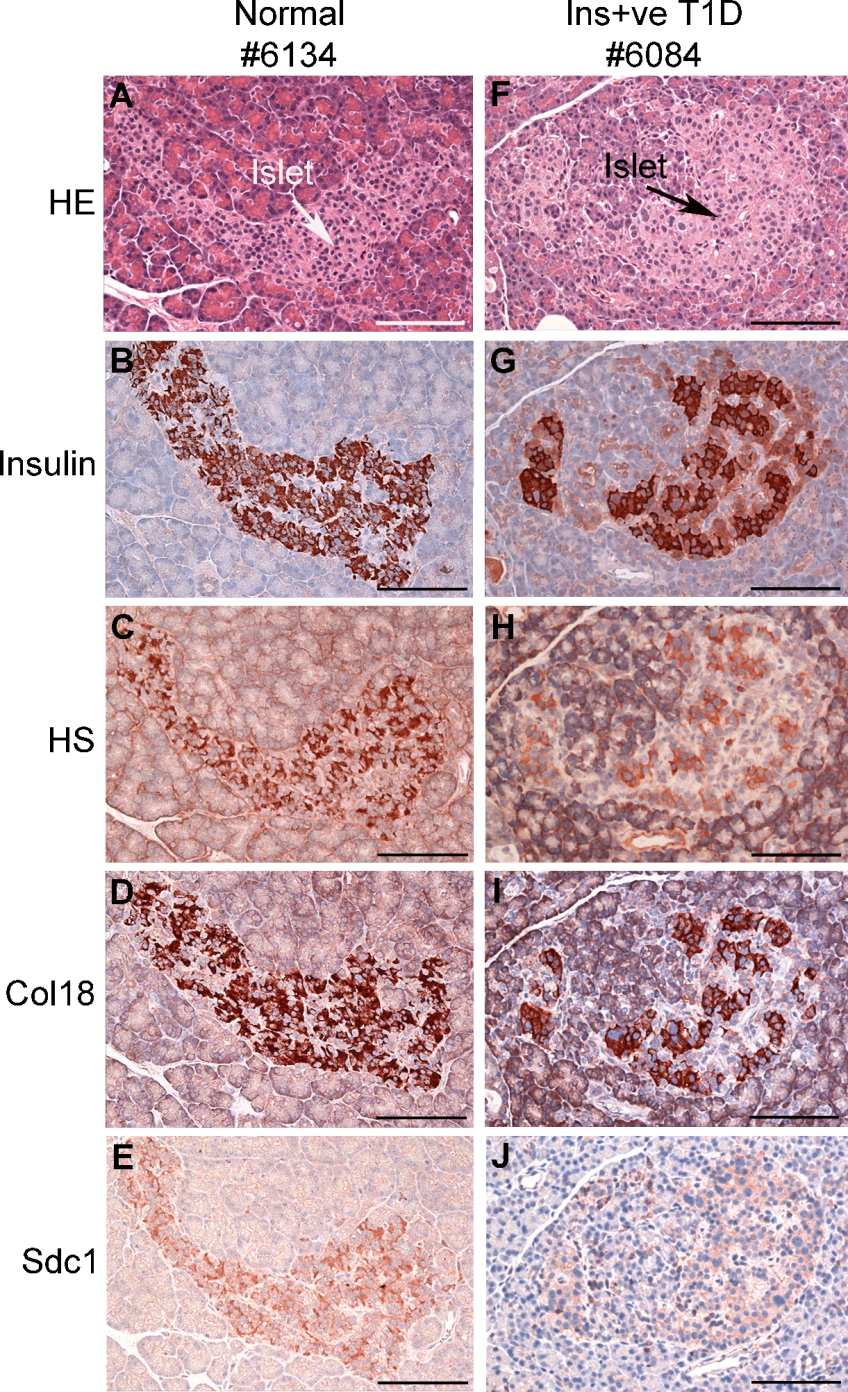
Intra-islet HS and HSPG core proteins in human pancreases correlate with insulin-positive beta cells. Immunohistochemical analyses of (**A-E**) a normal human pancreas (nPOD #6134) and (**F-J**) a pancreas with insulin-containing (Ins+) islets from a donor with T1D (nPOD #6084, 4 years post-T1D onset) show the distribution of (**B,G**) insulin-positive beta cells, intra-islet (**C,H**) HS, (**D,I**) Col18 core protein and (**E,J**) Sdc1 core protein. (**A,F**), H&E. Scale bar = 100 μm.

**Fig 2:**
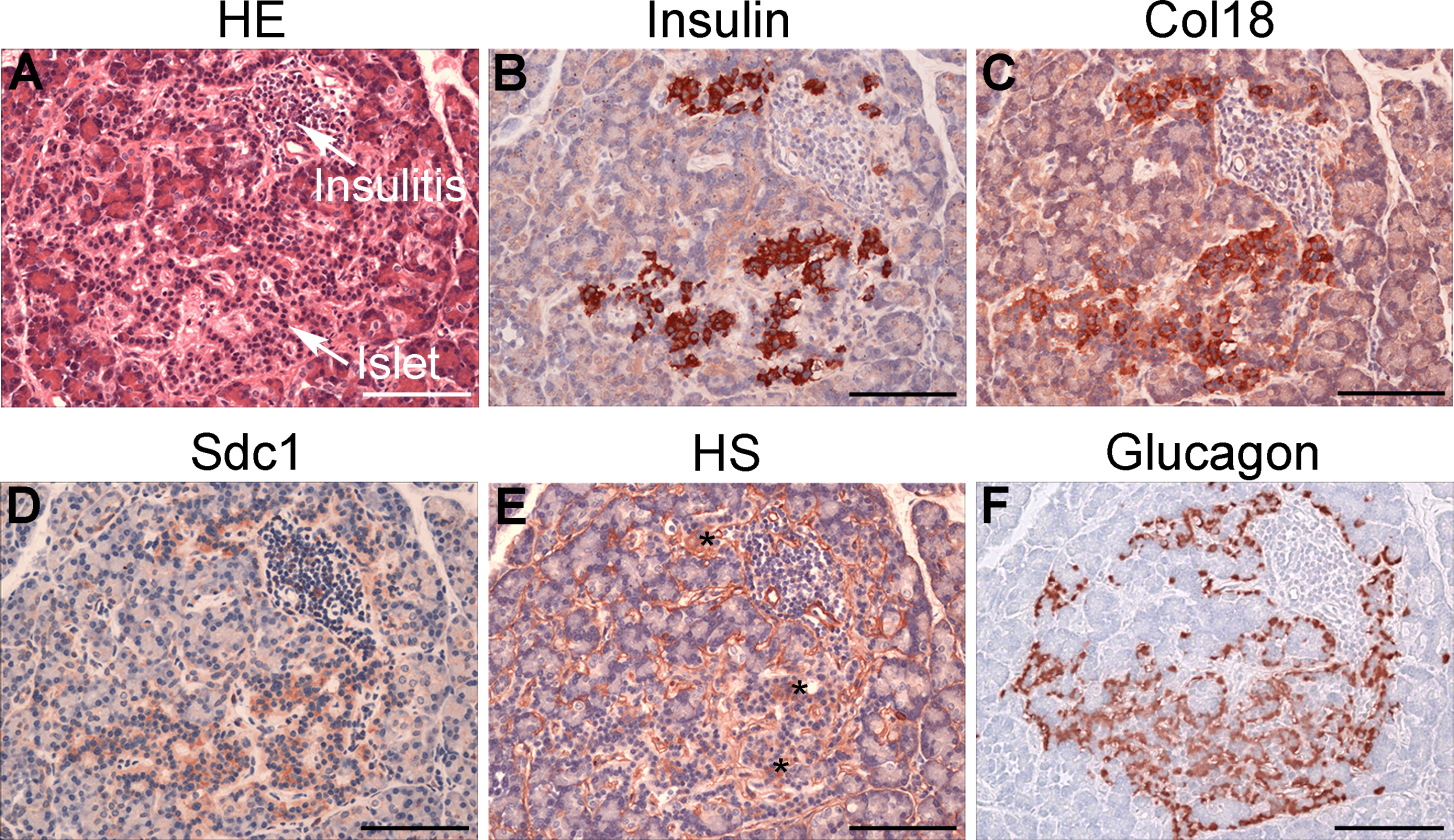
Immunohistochemical localization of intra-islet HS and HSPG core proteins in Ins+ T1D human pancreas with insulitis. Insulitis in the pancreas of nPOD #6070 (7 years post-T1D onset) (**A**) is adjacent to (**B**) residual insulin-positive beta cells which show staining for (**C**) Col18 and (**D**) Sdc1 core proteins but (**E**) little HS (*). (**F**) Glucagon staining is distinct from HSPGs (**C,D**) and HS (**E**). (**A**) H&E. Scale bar = 100 μm.

**Fig 3:**
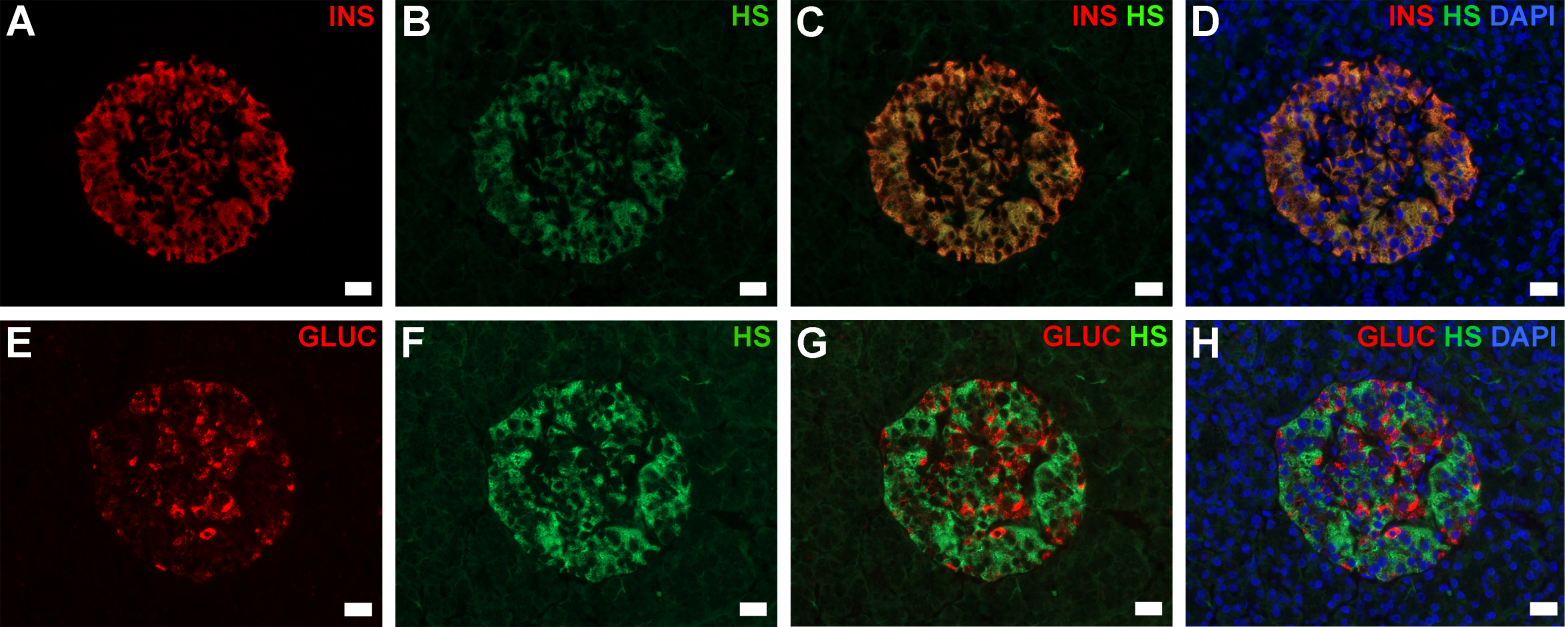
Intra-islet HS colocalizes with insulin not glucagon staining in normal human pancreas. Immunofluorescence staining of (**A**) insulin (INS), (**B**,**F**) HS and (**E**) glucagon (GLUC), in normal human pancreas (nPOD #6134). Nuclei were stained with DAPI (**D**,**H**). (**A)** anti-insulin Ab; (**B**,**F**) 10E4 anti-HS mAb; (**E)** anti-glucagon Ab; (**C,G**) merged (excluding DAPI); (**D**,**H**) merged (including DAPI). Scale bar = 20 μm.

**Fig 4:**
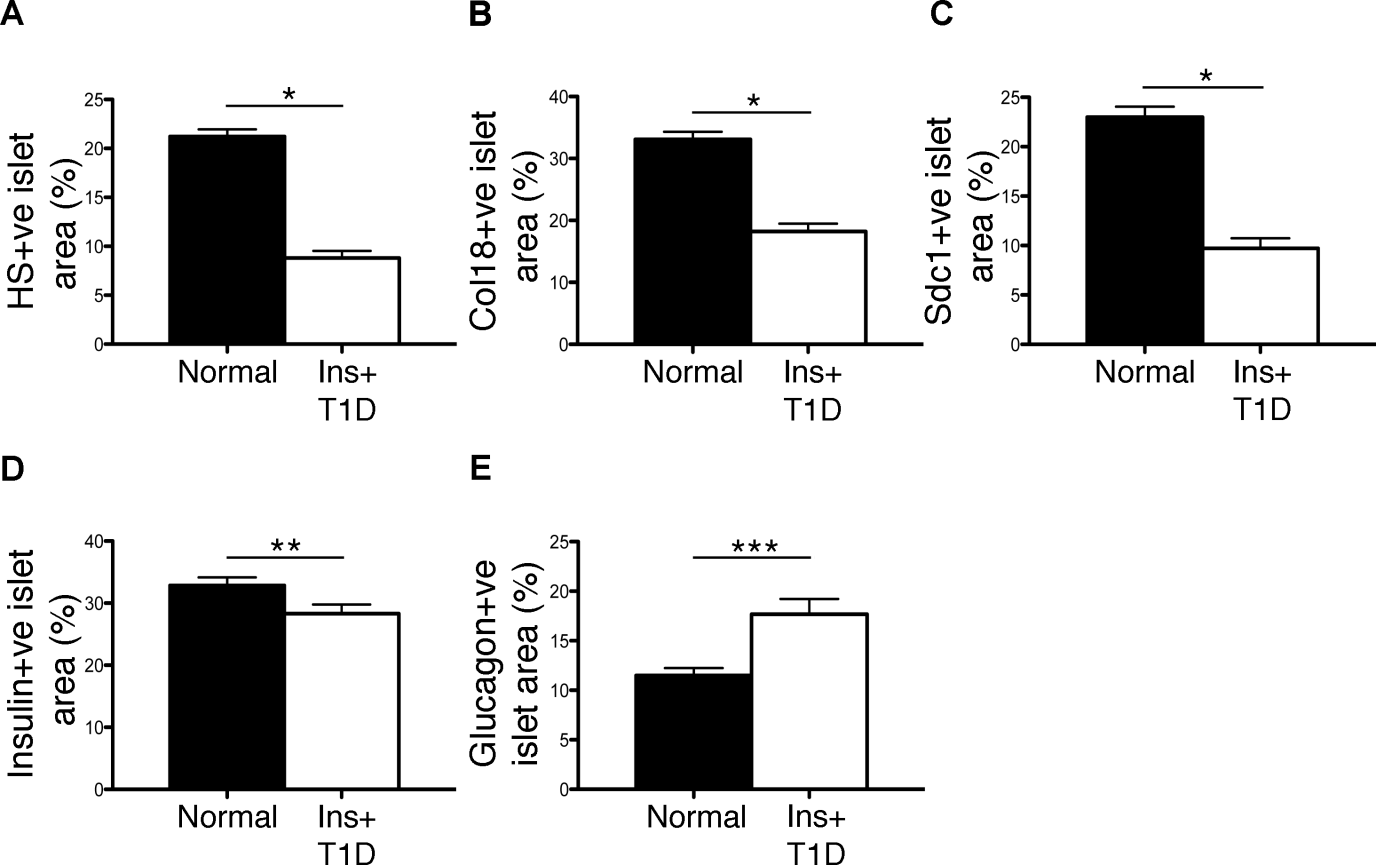
Intra-islet HS and HSPG core protein levels decline in Ins+ islets in human T1D. Morphometric analysis of the (**A**) HS-positive, (**B**) Col18-positive, (**C**) Sdc1-positive, (**D**) insulin-positive and (**E**) glucagon-positive islet area in normal human pancreases (black bars) and Ins+ T1D human pancreases (open bars). Data shows mean ± SEM; n = 6–8 pancreases (n = 56–80 islets) examined/group for normal controls and n = 6–8 pancreases (n = 52–66 islets)/group for Ins+ diabetic donors, except for analyses of Sdc1 where n = 4–5 pancreases (n = 40–42 islets) were examined/group. Significance was determined using unpaired Student’s t test, * = P<0.0001, ** = P = 0.0217 and Mann-Whitney test, *** P = 0.0025.

Immunohistochemistry revealed widespread intra-islet localization of HS as well as Col18 and Sdc1 core proteins in normal human pancreas, correlating with the distribution of the insulin staining (Fig 1A–1E). Similarly, in T1D pancreases with Ins+ islets, staining for Col18, Sdc1 and HS correlated with residual insulin-containing beta cells (Fig 1F–1J), a finding which was also observed in islets with insulitis (Fig 2). Immunofluorescence microscopy demonstrated that HS (identified by 10E4 mAb) co-localized with insulin (Fig 3A–3D) and not glucagon (Fig 3E–3H) in normal islets. Little or no staining for HS was observed in pseudoatrophic (insulin-negative, glucagon-positive) T1D islets (data not shown). Morphometric analyses of normal pancreas specimens revealed that the % islet area stained for HS, Col18 and Sdc1 was 21.2±0.7% (Fig 4A), 33.1±1.2% (Fig 4B), 23.0±1.1% (Fig 4C), respectively. Beta cells and alpha cells were identified by the insulin-positive islet area (32.9±1.3%; Fig 4D) and glucagon-positive area of staining (11.5±0.8%; Fig 4E), respectively (S1 Table). In Ins+ T1D pancreases, the % area of insulin staining in the remaining islet tissue was 86% of normal islets (Fig 4D and S1 Table). However, the area stained for HS, Col18 and Sdc1 was significantly reduced to 41% (P<0.0001), 55% (P<0.0001) and 42% (P<0.0001) of normal islets, respectively (Fig 4A–4C and S1 Table). In contrast, the glucagon-positive islet area increased 1.5-fold (Fig 4E and S1 Table). These data indicate that HS and HSPG core proteins were localized in the beta cells of normal human islets and that the loss of intra-islet HS during diabetes progression preceded the decline in insulin content.

### HS in isolated normal islets and beta cells

**Fig 5:**
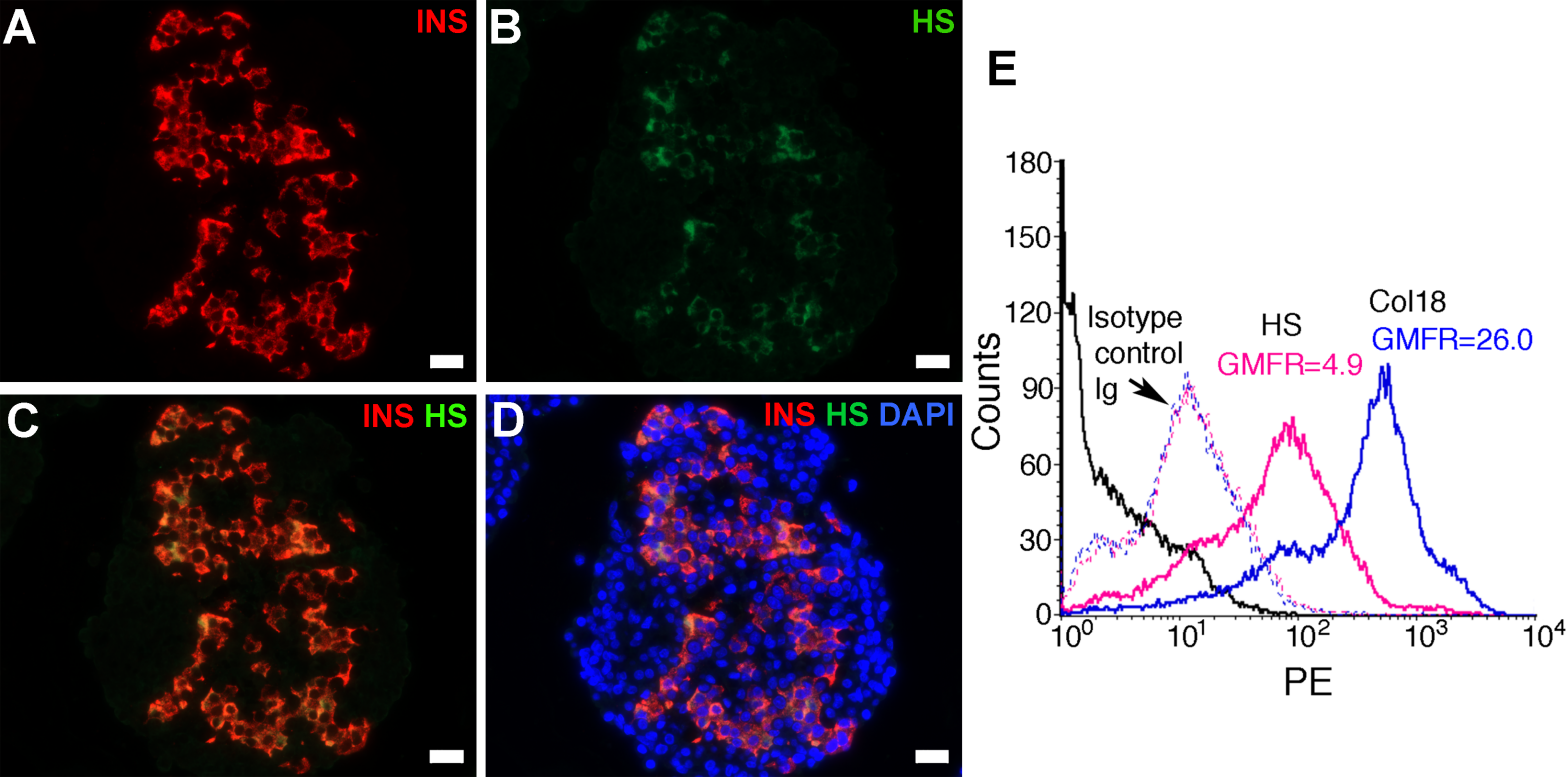
HS and HSPG core protein are localized inside isolated human islet beta cells. Immunofluorescence staining of 1 day-cultured isolated human islets show co-localization of (**A**) insulin (INS) and (**B**) HS. Nuclei were stained with DAPI (**D**). (**A)** anti-insulin Ab; (**B**) 10E4 anti-HS mAb; (**C**) merged (excluding DAPI); (**D**) merged (including DAPI). Scale bar = 20 μm. (**E**) Representative single color flow cytometry histograms of freshly isolated human islet cells (89% were NG-positive beta cells) show staining for intracellular HS (pink solid line) and Col18 (blue solid line) compared to background staining with corresponding isotype control Ig (dotted lines) and the autofluorescence of unstained cells (black solid line).

**Fig 6:**
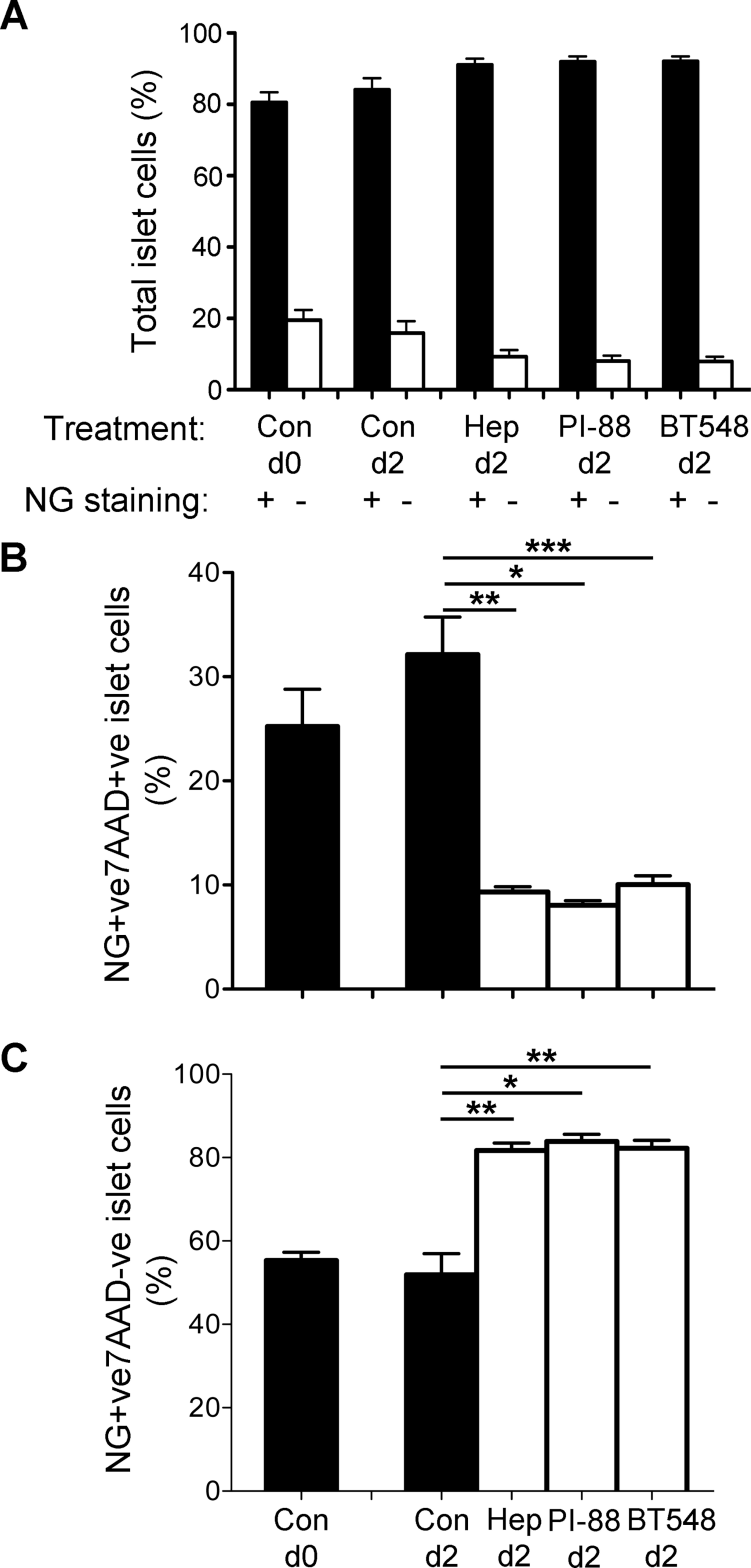
HS optimizes human beta cell survival *in vitro*. Flow cytometry analyses of the viability of freshly isolated human islet cells (Con) on day 0 and after culture for 2 days with or without heparin, PI-88 or BT548 at 50 μg/ml. (**A-C**) Islet cells were stained with Newport Green (NG) to identify beta cells and with 7AAD (**B** and **C**) to label non-viable cells; NG+ve, 7AAD-ve staining identified viable beta cells (**C**). Con, control; Hep, Heparin; BT548, chemically modified LMWH. Data (% islet cells) shows mean ± SEM; n = 8–10 independent experiments and significance was measured by non-parametric ANOVA (Kruskal-Wallis test) with Dunn’s Multiple Comparisons test. * P<0.001, **P<0.01, ***P<0.05.

In contrast to islets in normal human pancreas, immunofluorescence microscopy showed that insulin-positive staining in isolated normal human islets correlated with a wide variation in HS staining (Fig 5A–5D). These findings are consistent with partial loss of intra-islet HS occurring during the isolation of human islets, like isolated mouse islets [27, 28]. Flow cytometry analysis of dispersed islet cells revealed that levels of intracellular HS (mean GMFR = 3.6±0.3; n = 8 experiments) and Col18 (mean GMFR = 15.1±2.6; n = 7 experiments) were 2.6-fold (P = 0.0003) and 7.2-fold (P = 0.0012) higher, respectively, than on the surface of islet cells (Fig 5E, Table 1 and S2 Table). NG-positive staining revealed that 80.5±2.9% of human islet cells were beta cells (Fig 6A and S3 Table); in separate flow cytometry studies, 21%-24% of islet cells stained positively for intracellular glucagon i.e., alpha cells (data not shown). These findings further support the intracellular localization of HS and Col18 core protein inside human beta cells and suggest that HS levels in human beta cells decline during their isolation *in vitro*.

**Table 1:** Expression of HS and Col18 in freshly isolated human beta cells and after culture in the absence or presence of heparin.

	HS^a^		Col18^a^	
Treatment^b^	Intracellular	Cell Surface	Intracellular	Cell Surface
Control d0^c^	3.6±0.3	*1.4±0.1	15.1±2.6	^#^2.1±0.2
Control d2^d^	4.0±0.4	*2.5±0.3	9.5±1.4	2.8±0.4
Heparin d2^e^	3.3±0.4	1.8±0.2	11.5±1.7	^#^4.3±1.0

^a^ Flow cytometry GMFI data (see Methods) for intracellular (IC) and cell surface (CS) staining presented as mean ± SEM, n = 5–8 independent experiments.

^b^ Control d0, freshly dispersed control human islet cells were 83.7±9.4% NG-positive beta cells (n = 6 experiments); Control d2, 2 day cultured control human islet cells; Heparin d2, human isolated islet cells cultured for 2 days with 50 μg/ml heparin.

*P<0.01, One-way ANOVA with Bonferroni Multiple Comparison test

^#^P<0.05, Non-parametric One-way ANOVA (Kruskal-Wallis test) with Dunn’s Multiple Comparisons test

^c^ HS, IC versus CS, P = 0.0003, Mann-Whitney test; Col18, IC versus CS, P = 0.0012, Mann-Whitney test test

^d^ HS, IC versus CS, P = 0.0125, Unpaired t-test; Col18, IC versus CS, P = 0.0025, Mann-Whitney test

^e^ HS, IC versus CS, P = 0.0109, Mann-Whitney test; Col18, IC versus CS, P = 0.0089, Unpaired t-test

### Protection of human beta cell viability by HS replacers

Flow cytometry analyses showed no significant change in the beta cell population after culture of human islet cells for 2 days (84.1±3.3%; Fig 6A and S3 Table). On day 0, 25.2±3.6% of the total islet cell population were damaged or non-viable beta cells (NG-positive, 7AAD-positive; Fig 6B and S4 Table) and 55.3±2.0% were viable beta cells (NG-positive, 7AAD-negative; Fig 6C and S4 Table). Culture of the beta cells with the HS mimetics heparin, PI-88 and BT548 significantly improved beta cell viability by ~1.6-fold (P<0.001; Fig 6C and S4 Table) and significantly reduced the proportion of damaged/non-viable beta cells from 32.1±3.6% to 9.3±0.5% (P<0.01), 8.0±0.5% (P<0.001) and 10.1±0.8% (P<0.05), respectively (Fig 6B, S1 Fig and S4 Table). The minor population of NG-negative, 7AAD-positive cells also improved their survival after co-culture with HS mimetics (S2 Fig and S4 Table), suggesting that they could represent insulin-depleted beta cells. Beta cells cultured with heparin showed no detectable increase in intracellular HS (Table 1) due to the specificity of the 10E4 mAb for HS and not heparin [47]. In addition, human beta cells cultured with FITC-heparin for 1 day showed intracellular uptake of fluorescent heparin by confocal microscopy and protection from dying (S3 Fig). Together these findings indicate that exogenous HS mimetics promote the survival of human beta cells by acting as replacers for intracellular HS lost during islet/beta cell isolation.

### HS replacement protects human beta cells against oxidative damage

**Fig 7:**
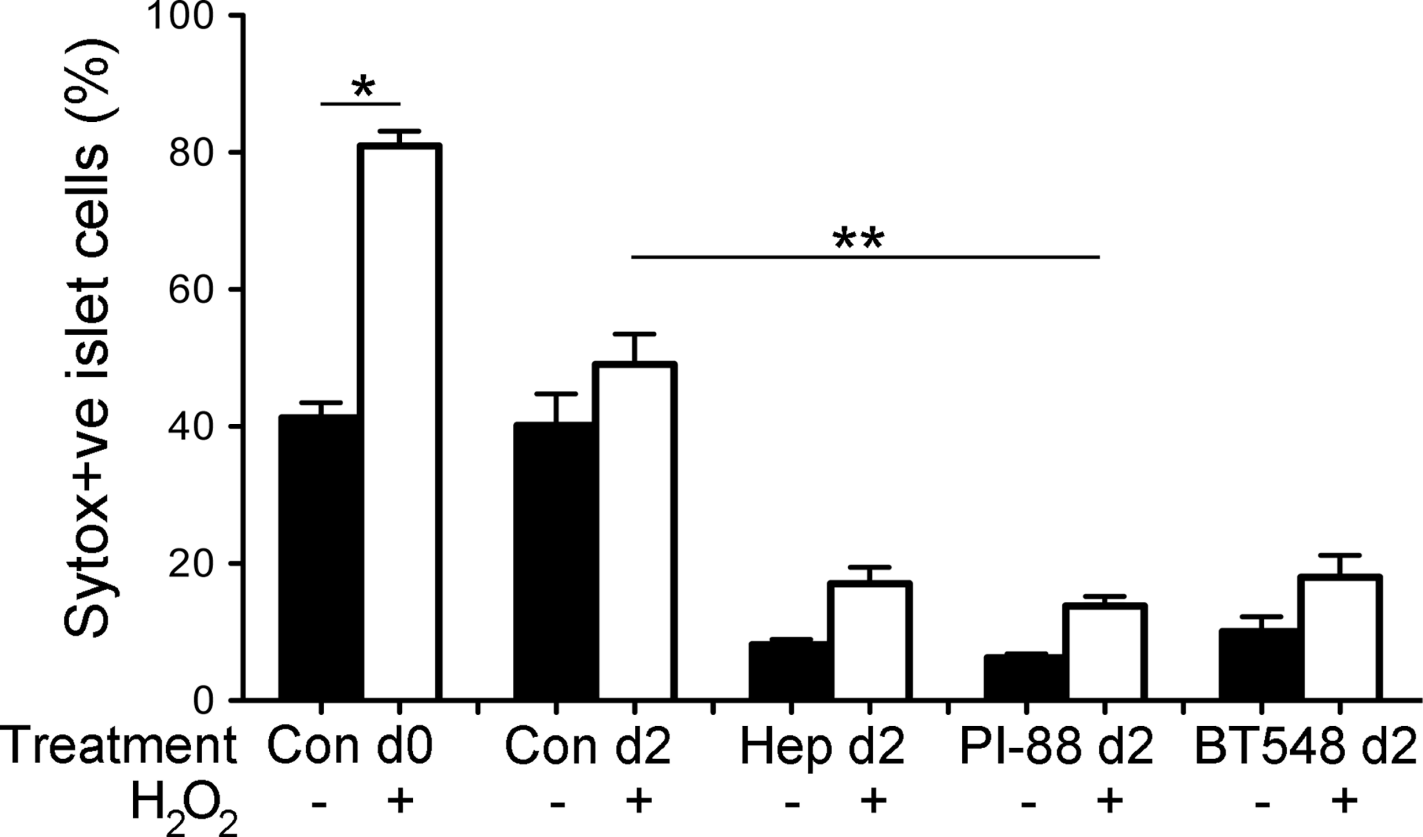
HS mimetics protect human beta cells from oxidative damage. Flow cytometric analyses of isolated human islet cells cultured with or without HS mimetics for 2 days and then treated acutely with hydrogen peroxide. Islet cell death/damage was measured by Sytox green fluorescence. Con, control; Hep, Heparin; BT548, chemically modified LMWH. Data shows mean ± SEM; n = 11–12 independent experiments. Con d0 versus Con d0 + hydrogen peroxide, Unpaired t-test, *P<0.0001; Con d2 + hydrogen peroxide versus PI-88 d2 + hydrogen peroxide, non-parametric ANOVA with Dunn’s Multiple Comparisons test, ** P<0.05.

The improved viability of human islet cells after HS replacement was also observed by flow cytometry analysis of Sytox green uptake (6.2–10.1% cell damage/death versus 40.2% for controls; Fig 7 and S5 Table). Freshly isolated human beta cells treated with hydrogen peroxide (source of reactive oxygen species (ROS)), showed a 2-fold increase in cell death, marked by an increase in the proportion of Sytox green-positive cells from 41.3±2.2% to 80.9±2.2% (Fig 7 and S5 Table). In contrast, culture with heparin, PI-88 or BT548 for 2 days and subsequent treatment with hydrogen peroxide substantially decreased the Sytox green-positive non-viable human beta cells to 13.8%-18.0% of total cells, compared to 49.1% for corresponding controls (Fig 7 and S5 Table). Beta cell survival after exposure to hydrogen peroxide was significantly improved after HS replacement with PI-88 (P<0.05).

### Heparanase is expressed by islet-infiltrating leukocytes in human diabetes

**Fig 8:**
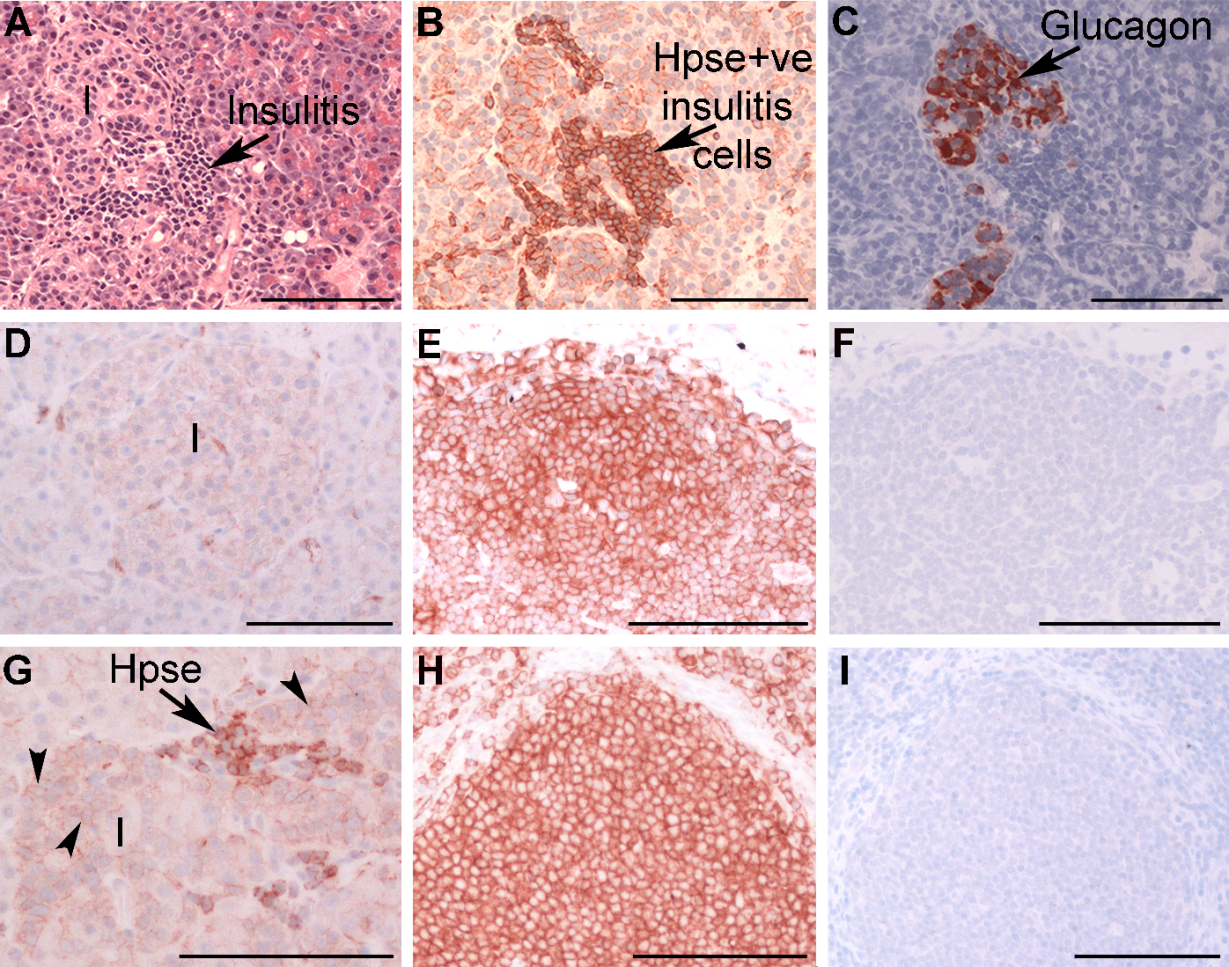
Heparanase is expressed by human insulitis leukocytes. (**A**) An islet (I) with insulitis in nPOD pancreas #6084 (4 years post-T1D onset) shows (**B**) leukocytes strongly expressing heparanase (Hpse) and (**C**) glucagon staining. Normal human islet cells (nPOD #6012) (**D**) showed negligible heparanase expression compared to pancreatic lymph node (PLN) from the same donor (**E**). Islet cells in Ins+ T1D pancreas (nPOD #6070) weakly expressed heparanase (arrowhead), compared to nearby infiltrating leukocytes (arrow) (**G**) and host PLN (**H**). Background staining with isotype control Ig was absent in PLNs (**F** and **I**). (**A**) H&E; (**B, D, E, G, H**) anti-Hpse HP130 mAb; (**C**) anti-glucagon mAb; (**F, I**) mouse IgM. Scale bar = 100 μm.

Consistent with a significant decline in the HS content of Ins+ diabetic human islets, islet-infiltrating leukocytes (Fig 8A–8C) showed intense cell surface expression of the HS-degrading endoglycosidase heparanase. In comparison, islet cells in normal and diabetic pancreases showed negligible or weak expression, respectively (Fig 8D and 8G). These findings suggest that like NOD mice [27], HS in human beta cells may be degraded by heparanase produced by islet-infiltrating leukocytes.

## Discussion

This study demonstrates that HS (identified by 10E4 mAb) and the HSPG core proteins for Col18 and Sdc1 are strongly expressed in normal human islets, and importantly, are localized inside beta cells. Our findings support a preliminary report of the immunolocalization of highly sulfated HS in insulin-containing beta cells of a single human pancreas [29]. Consistent with the finding of less-sulfated HS in alpha cells [29], we confirm that highly sulfated HS (recognized by 10E4 mAb) is selectively expressed in normal human beta cells and is absent in alpha cells. We previously reported that the uptake of highly sulfated but not under-sulfated HS protected mouse beta cells against dying in culture or from oxidant-induced death [27]. Using highly sulfated HS mimetics as HS replacers, we have now confirmed that intracellular HS plays a critical role in the viability of human beta cells. This key function underpins the unique localization of highly sulfated HS in healthy normal beta cells.

From a mechanistic standpoint, we propose that intracellular HS normally preserves beta cell viability *in situ* by acting as a constitutive nonenzymatic antioxidant, providing immediate protection from damaging reactive chemical species (e.g., ROS) generated during normal metabolism [27]. Our mouse studies have revealed, however, that HS loss during islet isolation is due, at least in part, to the generation of excessive levels of ROS, which can depolymerize HS [27, 28, 48, 49]. Furthermore, we have shown that islet HS is not readily repaired by *de novo* synthesis *in vitro* [28]. In human beta cells, the expression of ROS scavenging enzymes (e.g., superoxide dismutase 2 (SOD2)) can be induced [50], a process that may represent an alternate mechanism for neutralizing elevated levels of ROS during culture. We observed that *in vitro*, HS replacement in human beta cells better preserves their survival and is more protective against oxidative damage than alternative mechanisms that operate or are induced in control beta cells (see Fig 7). While the subcellular sites of endogenous HS and HSPGs in beta cells have yet to be defined, the localization of FITC-labelled heparin in the cytoplasm (S3C Fig) correlated with improved viability (S3B Fig). These findings further suggest that preserving islet HS during human islet isolation could improve the viability of transplanted islets in the peri-transplant period [51, 52], and in conjunction with optimal combinations of anti-rejection strategies [51–53] could improve islet graft outcomes for T1D patients.

We investigated whether the depletion of beta cell HS correlates with the progression of T1D in humans, as in NOD mice [27]. We found that the HS and HSPG core protein profiles of insulin-positive islets showed a profound decline in the pancreas of organ donors with T1D, compared to non-diabetic islets. Based on the role for HS in human beta cell survival and the striking protection from ROS *in vitro*, we postulate that the loss of HS observed in T1D pancreases primes beta cells for oxidative damage and impairs their viability. Supporting this notion, there is increasing evidence of beta cell dysfunction in T1D [54] and this likely precedes autoimmune-mediated beta cell death. Such changes in beta cell viability/function could also impact nearby alpha cells, possibly leading to their expansion (see Fig 4E) or redistribution within the islets, as reported previously in a mouse model of diabetes [55]. Overall, our findings strongly suggest that the level of intracellular HS represents a robust marker of the viability of residual beta cells during T1D progression.

Significantly, in human T1D, we observed that beta cells lose HS before insulin. We also found that ~15% of the total Ins+ islets showed insulitis, confirming the low frequency reported by other studies [11, 56]. In both NOD mice and man, immunofluorescence microscopy has demonstrated that infiltrating leukocytes destroy the peri-islet BM at the time they invade the islet tissue [20, 26]. Korpos et al reported that human leukocytes express ECM-degrading proteases (cathepsins) that solubilize the islet BM [20]. Importantly, in this study, we provide evidence for strong expression of cell surface heparanase, a HS-degrading enzyme, by human insulitis leukocytes. It should be noted, however, that although similar heparanase staining was observed for lymph node leukocytes, the anti-heparanase antibody used for immunohistochemistry did not distinguish between inactive (latent or proenzyme) and catalytically active enzyme [27, 34]. As previously found during T1D development in NOD/Lt mice [27], heparanase produced by insulitis leukocytes plays a critical role in degrading HS in the islet BM and inside beta cells, rendering the beta cells highly susceptible to oxidant-mediated damage and death. Additionally, leukocyte-derived proteases could contribute to the destruction of HSPG core proteins in beta cells, a process that may require initial cleavage of their HS chains. It is possible that the relative contribution of destructive mediators by different types of leukocytes could contribute to the age-related heterogeneity of the pathogenesis of T1D [2, 18, 57]. If multiple mechanisms operate, combined therapeutic strategies are likely to be required for impeding disease progression. Moreover, where islet HS is lost in the absence of insulitis, beta cell HS could potentially be degraded by heparanase produced by the beta cells themselves or possibly by high levels of endogenous ROS. Interestingly, in acute pancreatitis in mice and colitis in mice and humans, heparanase expression is strongly induced or elevated in pancreatic acinar cells [37] and gut epithelial cells [38], respectively, and contributes to local inflammation. The differences between these findings and our T1D study could be related to islet tissue-specific properties, differences in the relative contribution of adaptive and innate immune responses and/or to the different anti-heparanase antibodies (recognizing latent and/or catalytically active enzyme) and antigen retrieval methods used. Of significance for human T1D, we identify intracellular HS in beta cells as a requirement for beta cell viability and as a target for early destruction during disease progression.

We propose that at the time of T1D diagnosis, the survival of residual beta cells could potentially be maintained by HS replacement and/or HS preservation via blockade of heparanase activity. Importantly, HS replacers such as heparin, PI-88 and BT548 profoundly inhibit the catalytic activity of recombinant human heparanase (S4 Fig), suggesting that as dual activity drugs, they could represent a new class of therapeutic for restoring beta cell HS and blunting or preventing the progression of T1D. In summary, intracellular HS plays an important role in the viability of human beta cells. This study shows that HS levels are a sensitive marker of beta cell health (high HS) and deterioration (low HS) during T1D progression and suggest that HS preservation therapy could represent a novel aid for beta cell rescue.

## Supporting information

S1 Appendix Methods for supporting information. (DOCX)

S1 Fig HS replacement using HS mimetics improves the viability of human beta cells. Representative flow cytometric data shows the viability of control human beta cells on day 0 (top panel) and day 2 (bottom panel) after staining with Newport Green (NG) and 7AAD (i.e., NG+ve, 7AAD-ve) and a striking > 2-fold improvement in beta cell viability (upper left quadrant; bottom panel) after culture with 50 μg/ml HS mimetic (heparin, PI-88, or BT548 (chemically modified LMWH)) for 2 days.
(TIF)

S2 Fig HS replacement improves insulin-negative human islet cell survival *in vitro*. Flow cytometry analyses of islet cell viability following Newport Green (NG) and 7AAD staining of human islet cells cultured for 2 days with HS mimetics heparin, PI-88 or BT548 at 50 μg/ml (from Fig 6 and S4 Table) shows (**A**) a 6.6–8.5-fold decrease in NG-ve7AAD+ve islet cells and (**B**) no significant change in NG-ve7AAD-ve islet cells i.e., viable non-beta cells. The minor population of NG-ve7AAD+ve control cells at d0 (Con, d0) may represent insulin-depleted beta cells which are rescued by HS reconstitution during culture for 2 days with HS replacers. Con, control; Hep, Heparin; BT548, chemically modified LMWH. Data (% islet cells) shows mean ± SEM; n = 8–10 independent experiments. Significance was analyzed by non-parametric ANOVA (Kruskal-Wallis Test) with Dunn’s Multiple Comparisons test, ** = P<0.01, *** = P<0.05.
(TIF)

S3 Fig Uptake of FITC-heparin by cultured human beta cells correlates with improved viability. Human beta cells cultured for 1 day (**A**) without or (**B**) with 50 μg/ml FITC-heparin were stained with 7AAD and examined by flow cytometry to determine the viability of control and FITC-heparin+ve beta cells. Percentage of total cells is shown in the quadrants. (**C**) In parallel, confocal microscopy of 1 day-cultured beta cells (from **B)**, confirmed the intracellular uptake of FITC-heparin (green fluorescence) and its accumulation predominantly in the cytoplasm. Immunofluorescence staining with DAPI (blue) identifies the nucleus in the isolated human islet cells.
(TIF)

S4 Fig Inhibition of human heparanase activity by HS mimetics. The activity of recombinant human heparanase was inhibited by (**A**) heparin (IC_50_ = 73.5 ng/ml (5.3 nM)), (**B**) PI-88 (IC_50_ = 79.8 ng/ml (33.2 nM)) and (**C**) BT548 (chemically modified LMWH; IC_50_ = 29.2 ng/ml (9.7 nM)), in a colorimetric assay using Fondaparinux as substrate.
(TIF)

S1 Table Percentage of islet area stained for HS, HSPGs (Col18 and Sdc1), insulin and glucagon in normal healthy and T1D human pancreases. (XLSX)

S2 Table Geometric mean fluorescence ratio values for HS and Col18 expression in human islet cells. (XLSX)

S3 Table Proportion of beta cells in isolated human islet cells ± culture with HS mimetic. (XLSX)

S4 Table Viability of human beta cells ± culture with HS mimetic. (XLSX)

S5 Table Viability of human islet cells ± culture with HS mimetic and with/without acute exposure to hydrogen peroxide. (XLSX)
