# Supplementary material for: Loss of intra-islet heparan sulfate is a highly sensitive marker of type 1 diabetes progression in humans
Source: PLoS One. 2018 Feb 7;13(2):e0191360. doi: 10.1371/journal.pone.0191360 (PMC5802856; doi:10.1371/journal.pone.0191360)
Supplement: S1 Appendix — (DOCX) [file pone.0191360.s001.docx]

**Supporting Information**

**S1 Appendix. Methods for supporting information.**

**Culture of human islet cells with FITC heparin.** Human islet cells were cultured with 50 μg/ml of FITC-labelled heparin [[1](#_ENREF_1)] for 1-2 days for studies of cellular uptake by flow cytometry and confocal microscopy. Uptake of FITC-heparin [[1](#_ENREF_1)] by cultured human islet cells was measured as the % FITC+ve cells. Cells were analyzed using a BD LSRI flow cytometer and CellQuest™ Pro software (version 6.0; BD Biosciences) as well as a LEICA confocal microscope (LEICA TCS SP5, Leica Microsystems CMS GmbH, Mannheim, Germany). Images were processed using Adobe Photoshop CS3, version 10.0.1.

**Heparanase activity assay** Heparanase (Hpse) activity was measured using a colorimetric assay [[2](#_ENREF_2)]. The reducing disaccharides formed by cleavage of Fondaparinux (a synthetic heparin pentasaccharide; Arixtra, GlaxoSmithKline, Boronia, VIC, Australia) by recombinant active human heparanase (R&D Systems, Minneapolis, MN) were detected using the tetrazolium salt WST-1 (4-[3-(4-iodophenyl)-2-(4-nitrophenyl)-2H-5-tetrazolio]-1,3-benzene disulfonate; Dojindo Molecular Technologies, Kumamoto, Japan) [[2](#_ENREF_2)]. The inhibition of recombinant Hpse activity (IC_50_) by heparin, PI-88 and BT548 (chemically modified LMWH; 5 - 600 nM) was determined by measuring the optical density at 584 nm using a plate reader (Tecan, Infinite M200 Pro, Maennedorf, Switzerland).

**References for S1 Appendix**

1. Ziolkowski AF, Popp SK, Freeman C, Parish CR, Simeonovic CJ. Heparan sulfate and heparanase play key roles in mouse beta cell survival and autoimmune diabetes. J Clin Invest. 2012; 122(1): 132-141. PMID: 22182841.

2. Hammond E, Li CP, Ferro V. Development of a colorimetric assay for heparanase activity suitable for kinetic analysis and inhibitor screening. Anal Biochem. 2010; 396(1): 112-116. PMID: 19748475.
