# Supplementary figures and images for: Loss of intra-islet heparan sulfate is a highly sensitive marker of type 1 diabetes progression in humans
Source: PLoS One. 2018 Feb 7;13(2):e0191360. doi: 10.1371/journal.pone.0191360 (PMC5802856; doi:10.1371/journal.pone.0191360)

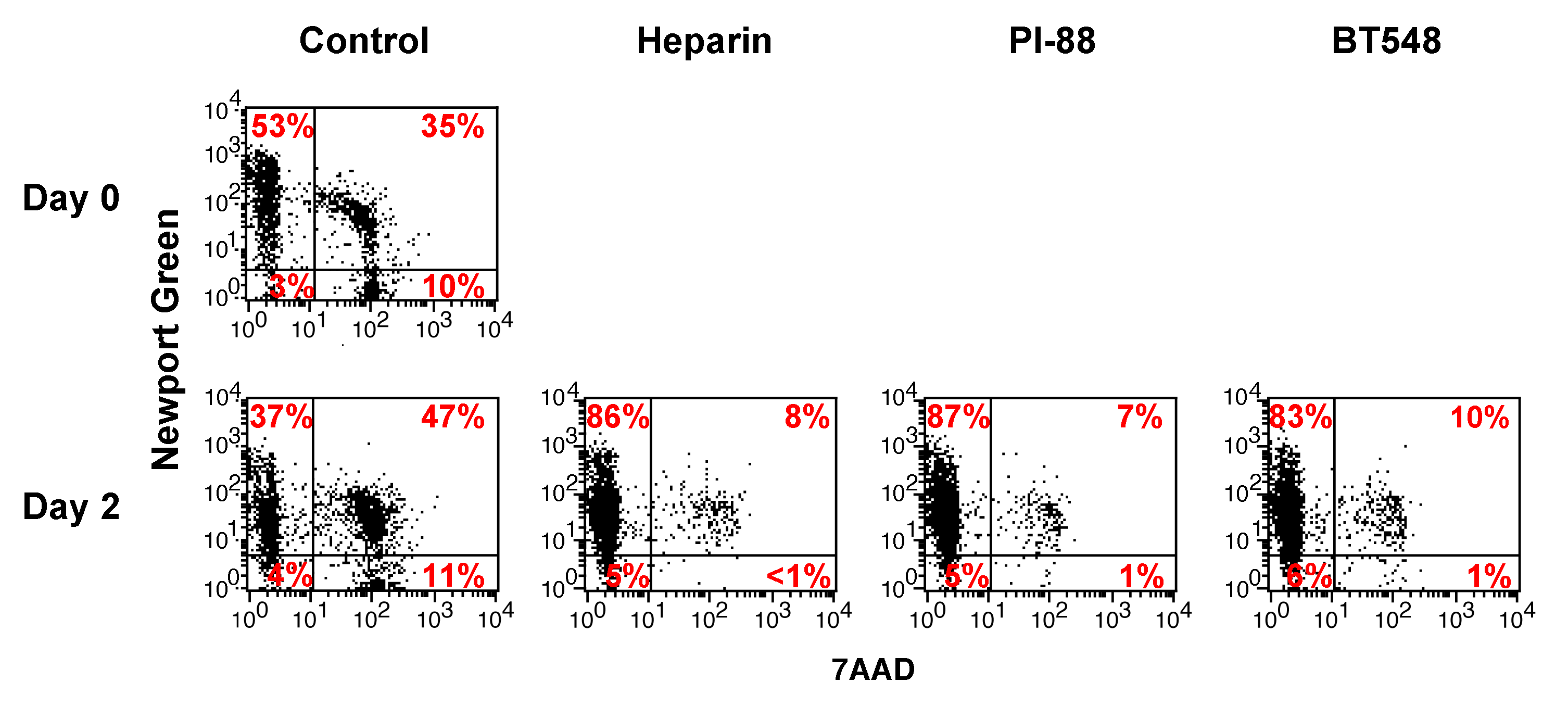

Supplement: S1 Fig — Representative flow cytometric data shows the viability of control human beta cells on day 0 (top panel) and day 2 (bottom panel) after staining with Newport Green (NG) and 7AAD (i.e., NG+ve, 7AAD-ve) and a striking > 2-fold improvement in beta cell viability (upper left quadrant; bottom panel) after culture with 50 μg/ml HS mimetic (heparin, PI-88, or BT548 (chemically modified LMWH)) for 2 days. (TIF) [file pone.0191360.s002.tif]

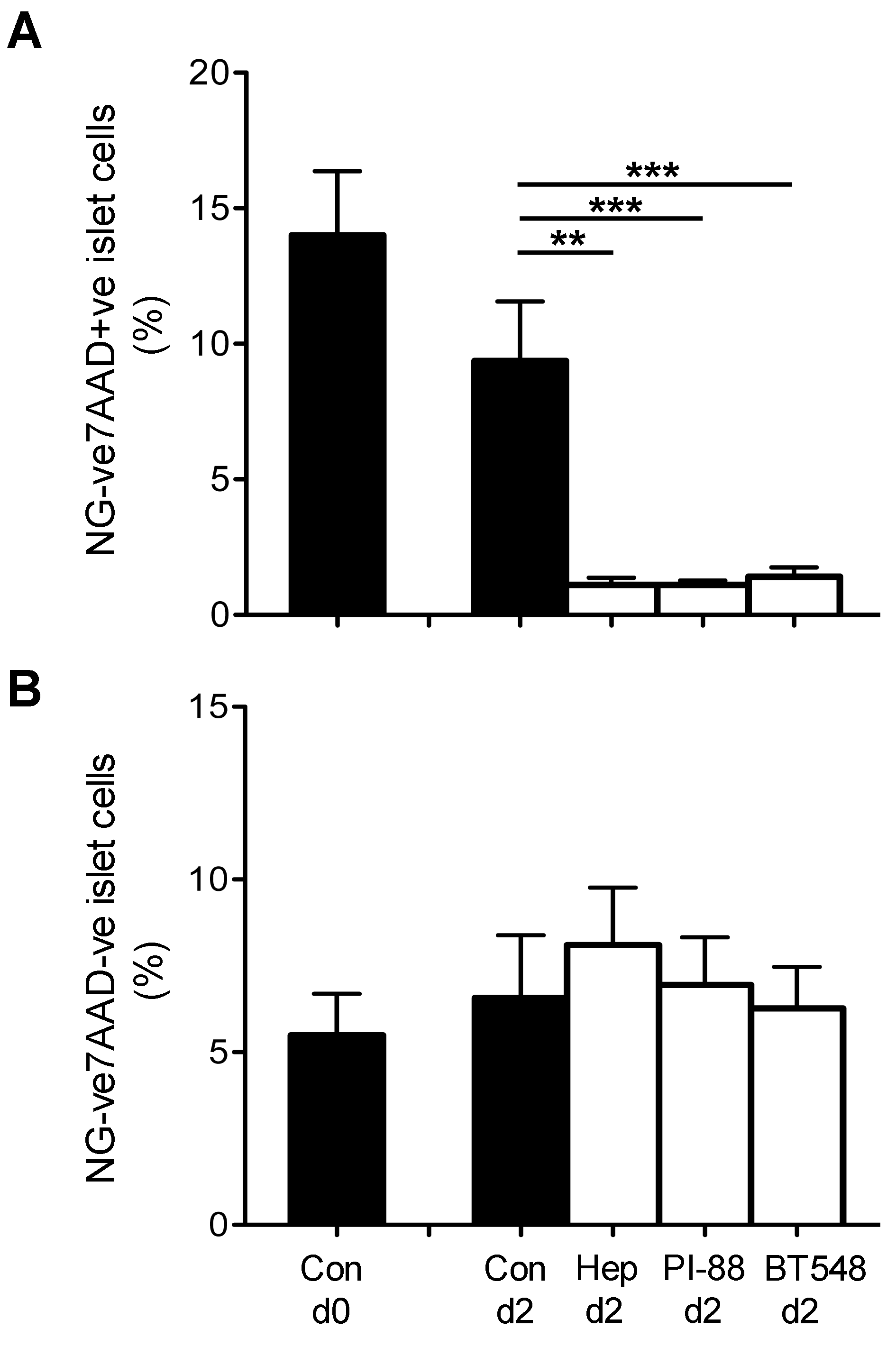

Supplement: S2 Fig — Flow cytometry analyses of islet cell viability following Newport Green (NG) and 7AAD staining of human islet cells cultured for 2 days with HS mimetics heparin, PI-88 or BT548 at 50 μg/ml (from Fig 6 and S4 Table) shows (A) a 6.6–8.5-fold decrease in NG-ve7AAD+ve islet cells and (B) no significant change in NG-ve7AAD-ve islet cells i.e., viable non-beta cells. The minor population of NG-ve7AAD+ve control cells at d0 (Con, d0) may represent insulin-depleted beta cells which are rescued by HS reconstitution during culture for 2 days with HS replacers. Con, control; Hep, Heparin; BT548, chemically modified LMWH. Data (% islet cells) shows mean ± SEM; n = 8–10 independent experiments. Significance was analyzed by non-parametric ANOVA (Kruskal-Wallis Test) with Dunn’s Multiple Comparisons test, ** = P<0.01, *** = P<0.05. (TIF) [file pone.0191360.s003.tif]

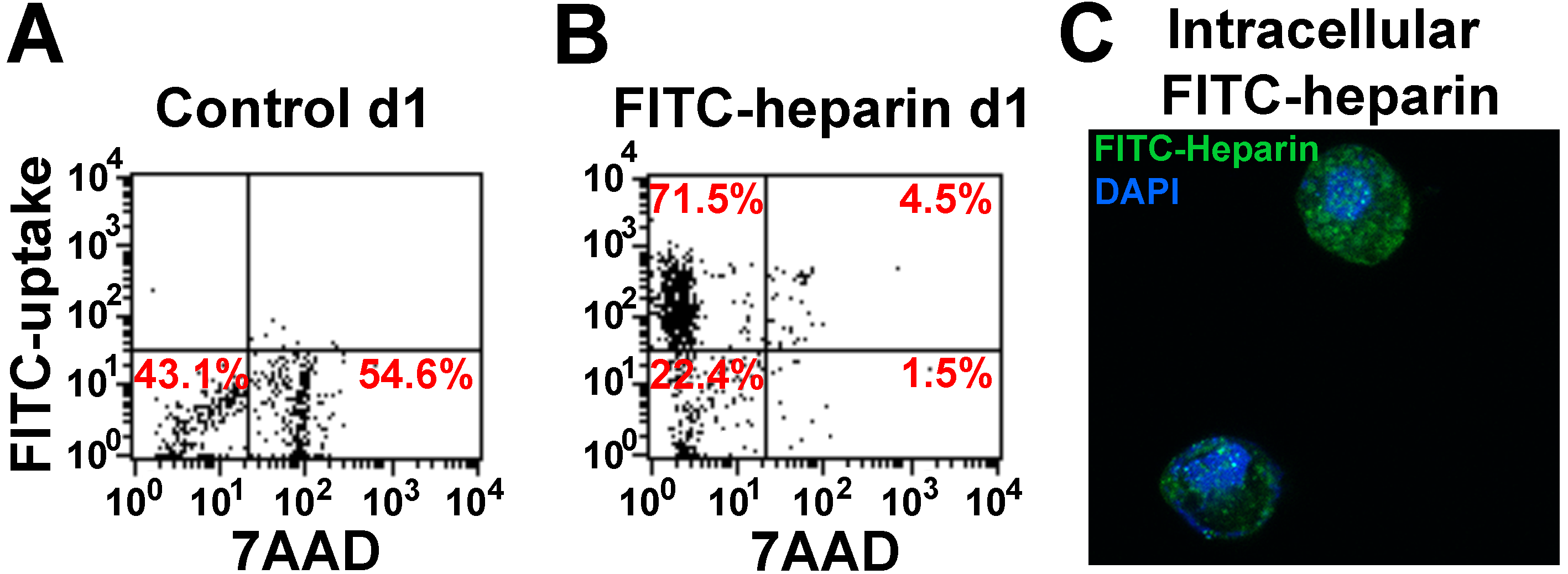

Supplement: S3 Fig — Human beta cells cultured for 1 day (A) without or (B) with 50 μg/ml FITC-heparin were stained with 7AAD and examined by flow cytometry to determine the viability of control and FITC-heparin+ve beta cells. Percentage of total cells is shown in the quadrants. (C) In parallel, confocal microscopy of 1 day-cultured beta cells (from B), confirmed the intracellular uptake of FITC-heparin (green fluorescence) and its accumulation predominantly in the cytoplasm. Immunofluorescence staining with DAPI (blue) identifies the nucleus in the isolated human islet cells. (TIF) [file pone.0191360.s004.tif]

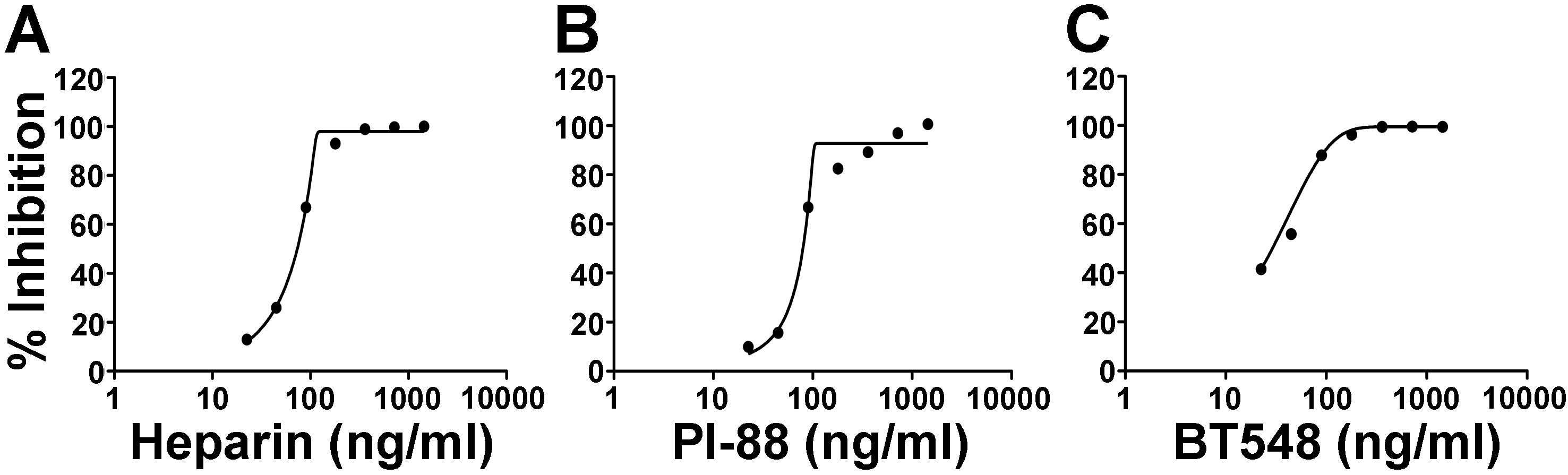

Supplement: S4 Fig — The activity of recombinant human heparanase was inhibited by (A) heparin (IC50 = 73.5 ng/ml (5.3 nM)), (B) PI-88 (IC50 = 79.8 ng/ml (33.2 nM)) and (C) BT548 (chemically modified LMWH; IC50 = 29.2 ng/ml (9.7 nM)), in a colorimetric assay using Fondaparinux as substrate. (TIF) [file pone.0191360.s005.tif]
